# Traditional knowledge and cultural importance of *Borassus aethiopum* Mart. in Benin: interacting effects of socio-demographic attributes and multi-scale abundance

**DOI:** 10.1186/s13002-018-0233-8

**Published:** 2018-05-15

**Authors:** Kolawolé Valère Salako, Francisco Moreira, Rodrigue Castro Gbedomon, Frédéric Tovissodé, Achille Ephrem Assogbadjo, Romain Lucas Glèlè Kakaï

**Affiliations:** 1 Laboratoire de Biomathématiques et d’Estimation Forestières, Faculté des Sciences Agronomiques, Université d’Abomey-Calavi, 04 BP 1525, Cotonou, Bénin; 2 REN Biodiversity Chair, CIBIO/InBIO – Centro de Investigação em Biodiversidade e Recursos Genéticos, Universidade do Porto, Campus Agrário de Vairão, 4485-601 Vairão, Portugal; 3 CEABN/InBIO – Centro de Ecologia Aplicada “Professor Baeta Neves”, Instituto Superior de Agronomia, Universidade de Lisboa, Tapada da Ajuda, 1349-017 Lisbon, Portugal; 4 Laboratoire d’Ecologie Appliquée, Faculté des Sciences Agronomiques, Université d’Abomey-Calavi, 03 BP 1974, Cotonou, Bénin

**Keywords:** Ethnobotany, Knowledge distribution, Wild palm, Conservation, *Borassus aethiopum* Mart., Benin

## Abstract

**Background:**

Eliciting factors affecting distribution of traditional knowledge (TK) and cultural importance of plant resources is central in ethnobiology. Socio-demographic attributes and ecological apparency hypothesis (EAH) have been widely documented as drivers of TK distribution, but their synergistic effect is poorly documented. Here, we focused on *Borassus aethiopum*, a socio-economic important agroforestry palm in Africa, analyzing relationships between the number of use-reports and cultural importance on one hand, and informant socio-demographic attributes (age category and gender) on the other hand, considering the EAH at multi-scale contexts. Our hypothesis is that effects of socio-demographic attributes on use-reports and cultural importance are shaped by both local (village level) and regional (chorological region level) apparency of study species. We expected so because distribution of knowledge on a resource in a community correlates to the versatility in the resource utilization but also connections among communities within a region.

**Methods:**

Nine hundred ninety-two face-to-face individual semi-structured interviews were conducted in six villages of low versus high local abundance of *B. aethiopum* spanning three chorological regions (humid, sub-humid and semi-arid) also underlying a gradient of increasing distribution and abundance of *B. aethiopum*. Number of use-reports and score of importance of uses of *B. aethiopum* were recorded in six use-categories including medicine, food, handcraft, construction, firewood, and ceremonies and rituals. Data were analyzed using Poisson and ordered logistic models.

**Results:**

Informants listed 121 uses for *B. aethiopum*: medicine (66 uses), handcraft (16 uses), food (16 uses), construction (12 uses), firewood (6 uses), and ceremonies and rituals (5 uses); but food use was the most culturally important use (2.45 ± 0.03), followed by construction (0.61 ± 0.03), medicinal (0.57 ± 0.03) and handcraft (0.56 ± 0.03), firewood (0.29 ± 0.02), and ceremonies and rituals (0.03 ± 0.01). Food use was the most important for women who were specialized in hypocotyls and fruits collection for commercialization. Men valued more the species for handcrafting, construction, and medicine. The number of use-reports was significantly dependent on age category and gender, and differences between age categories (young, adult, and old) in particular were dependent upon local and regional apparency. In particular, discrepancies among age categories were higher in areas of low abundance and distribution, which may be linked to different speed in the process of knowledge acquisition. In areas of low abundance, the species past abundance was also found instrumental in understanding current knowledge distribution.

**Conclusion:**

Findings suggest that studies aiming at understanding relationship between current TK and cultural importance of a resource on one hand and socio-demographic attributes on the other hand should consider the resource current local and regional apparency but further its local past abundance. The study also confirms that *B. aethiopum* is a socio-economic important species in Benin.

**Electronic supplementary material:**

The online version of this article (10.1186/s13002-018-0233-8) contains supplementary material, which is available to authorized users.

## Background

The overall trend of biodiversity loss [1] and the need to develop effective strategies for its conservation has led to emergence of several paradigms and principles of conservation. One of them, the principle of “conservation through use or trade” has been proposed as a key mechanism to provide incentives for the conservation of species and habitats by turning them into sources of income [2, 3]. The main idea is that conservation is more successful and livelihoods are improved when social and community beliefs and rights are understood and addressed in conservation programs [4]. This has resulted in increasing interests over local communities which in turn have led to a growing interest in the traditional knowledge (TK) they have on their environment [4, 5]. Ethnobotany, which aims at documenting interactions between humans and plants, has therefore become a core subject of conservation biology [6]. The need to better understand factors that determine spatial and intergenerational variation in TK has emerged, and quantitative tools are increasingly being developed to cope with the related issues [6].

Previous studies have shown that knowledge on the use of plant resources and its actual practices are a compounded effect of socio-demographic attributes, including people’s gender and age [4, 7, 8] with women and older people tending to have greater knowledge [8, 9]. Historical gender divisions of space and labor in households and societies [10], and increasing knowledge accumulation through time [6], have been often used respectively to explain such patterns.

In addition, the most highly available plants are more likely to be encountered, hence subject to greater experiment and, consequently, broader use and greater local importance [11]. This is referred as the ecological apparency hypothesis (EAH). Although this hypothesis has received mixed support in the literature [12, 13], partly because human culture is more complex to be influenced only by appearance [14], it is still central in ethnobotany [14].

The EAH has been often tested in studies dealing with the compared use of multiple species [15, 16] and rarely used for the study of a single species. In the latter case, the EAH predicts that people living in a landscape of high visibility (abundance) of a species have more knowledge of its uses than people in a landscape of low visibility. However, ecological apparency can be assessed at either local (e.g., village) or larger (e.g., chorological region) scale [17]. At local scale, ecological apparency hypothesis is expected to be a consequence of the direct contact between people and the used resource. However, at the regional scale, the amount of knowledge does not only result from the direct visibility of the species locally but also knowledge exchange between people of different communities/villages trough connectivity and networks. Whether and how local and global visibility of a species affect distribution of knowledge on its uses against age and gender has been poorly investigated. In an area of high abundance of a given resource, we might expect no or little difference of TK between age categories and to some extent between genders because the resource is so common that everyone knows and likely uses it. In contrast, in an area of low abundance, great discrepancies are predicted between both age categories and genders. Understanding such relationship is crucial for conservation biology in general and ethnobiology in particular. For example, this understanding would clarify on whether for documenting TK on a given taxon, sampling area of higher abundance is always better than area of lower abundance. In addition, it will also provide better insights in drivers of knowledge distribution in local communities.

There are several evidences that patterns of plant selection and use by local people, as well as the importance of plants, are driven by complex interactions of biophysical, social, cultural, cultual, political, and economic contexts [14, 18], including resource availability [19]; informant age, gender, and ethnic affiliation [20]; urbanization and informant education level [10, 21]; market importance, nutritive value, and number of complementary uses of species [22]; informant social network [23]; and taboos [24]. As such, TK on use of plants and their importance cannot be simplified to only resource abundance and socio-demographic attributes such as age and gender. The overarching goal of this study was rather to examine whether and how both local and regional apparency of a resource mediate gender- and age-related distribution of traditional knowledge on its uses as well as its cultural importance. To our knowledge, this issue has so far been little explored.

We used the case study of the palm species *Borassus aethiopum* Mart. in Benin, West-Africa. *B. aethiopum* is one of the most important wild palm species in West Africa [25]. Although the overall IUCN threat category of *B. aethiopum* is least concern (LC), the species suffers from local over-exploitation for palm wine and several other threats (http://www.iucnredlist.org/details/195913/0), hence considered threatened across its distribution range [26–29]. In Benin, *B. aethiopum* is found in traditional agroforestry systems and natural forests [30] but faces a serious threat of regeneration, and its populations are aging [26]. Based on IUCN Categories and Criteria of threat, *B. aethiopum* was categorized as “vulnerable” in Benin because of human disturbances on its populations [26].

Although several ethnobotanical reports exist on the species over its distribution range [28, 31–35], they are often narrowed to a locality or a small part of a country [35] and rarely used quantitative approaches to decipher complex interactions between humans and the species. Yet, such understanding could provide important baseline information for its sustainable use and management.

The main research question addressed here is whether (and how) local and regional abundance of *B. aethiopum* influences the relationships between TK and cultural importance on one hand and socio-demographic attributes (gender and age) on the other hand? We predicted that (1) TK and cultural importance of *B. aethiopum* increases with increased local and regional apparency, (2) TK and cultural importance of *B. aethiopum* are influenced by gender and age, and (3) both local and regional apparency influence the relationships between TK and cultural importance of *B. aethiopum* and socio-demographic attributes, with greater discrepancies between age and gender where the species is less apparent. The study also sought to document the countrywide uses and importance of *B. aethiopum* in Benin.

## Methods

### Study area

**Fig. 1:**
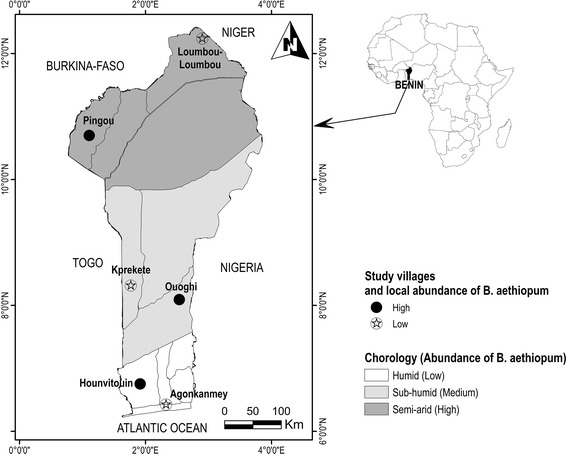
Map of the republic of Benin showing the three chorological regions and the study villages

The study was conducted in Benin (6° 25′ N–12° 30′ and 0° 45′ E–4° E), West Africa (Fig. 1) [36]. Benin is characterized by three contrasting chorological regions [37], northwards: the Guinean region (humid climate), the Sudan-Guinean region (sub-humid climate), and the Sudanian region (semi-arid climate) regions [38]. The country’s native vegetation has suffered severe degradation as a result of various intense anthropogenic activities. The vegetation becomes dominated by woodland and savannah northwards. The resident population in 2013 was nearly 10 million inhabitants, unequally distributed across the territory [39]. The population is mainly young (more than 40% are < 15 years old) and slightly female-biased (51.2%) [39]. The local economy is agriculture-based [39]. More than half the population (53.9%) live with less than 1 US dollars per day [40]. The average size of farmland per farmer is 1.7 ha, and more than 1/3 had less than 1 ha [41]. Regardless of socio-cultural groups, women have no or limited access to land [41].

### Study species

**Fig. 2:**
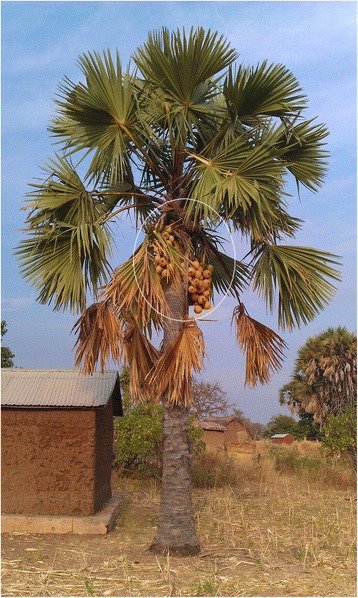
A female tree of *B. aethiopum* showing its fruits (white circle) in Northern Benin. Credits to Salako et al. [30]

The African fan palm, *B. aethiopum* (Fig. 2), belongs to family Arecaceae, subfamily Coryphoideae [42]. It is widespread and common across sub-Saharan Africa where it is a well-known and conspicuous component of savannas. *B. aethiopum* has a large and straight stem to 25 m tall and may reach 80-cm diameter. The fruits are massive, ovoid, and orange at maturity (Fig. 2). The mesocarp is pulpy and fragrant with many longitudinal fibers [25]. In Benin, *B. aethiopum* is found in all three chorological regions but with great differences in regard with distribution and abundance [30]. Overall, *B. aethiopum* becomes common with higher abundance northwards, but in all three regions, there exist areas of local low and high abundance [30].

### Sampling and data collection

In Benin, *B. aethiopum* has a wider distribution and larger abundance (trees ha^−1^) in the semi-arid region (24.54 ± 1.51) than in the sub-humid region (16.24 ± 1.79), and than in the humid region (7.06 ± 0.56) [30]. However, these chorological region patterns hide strong local differences within each region, where it is possible to identify areas with low and high local abundance. Based on previous studies [30], we therefore identified villages with high (> 20 adult trees ha^−1^) and low (up to 10 adult trees ha^−1^) local abundance of *B. aethiopum*. In each chorological region, we randomly selected one village with high abundance and one with low abundance (Fig. 1). Abundance at chorological regions was assumed as an indicator of the regional apparency, while abundance at village level represented local apparency. Following [43], three age categories were considered (age < 30 years for young; 30 ≤ age < 60 years for adults; age ≥ 60 years for old persons). Gender (men and women) was also considered. Primarily, it was planned to select in each village 30 informants for each combination of gender and age category, making a total of 1080 informants. But because of insufficient informants in some villages, 992 informants participated to the work (Additional file 1). Data were collected using face-to-face individual interviews based on a questionnaire (Additional file 2). The goal of the study in terms of gathering knowledge on *B. aethiopum* for a PhD research (this study was part of the PhD research of the first author on the conservation biology of *B. aethiopum* in Benin) was first explained to village authorities and next to each of the informant as to have their consent for participation before starting the interviews. Only individuals that consented to participate in the study were considered. Interviews were conducted with assistance of a local translator when necessary. The questionnaire comprised three main sections. The first was related to informant’s socio-demographic information (age, gender, and ethnic affiliations). The second consisted of a free-listing of the use-reports of *B. aethiopum*, defined as each specific use mentioned by the informant per plant part in the sense of [44]. For example if an informant mentioned use of fruits to cure malaria and the use of leaves to also cure malaria separately, we considered them as two separate use-reports. Use-reports were arranged per plant part in six use-categories adapted from [45]: food, handcraft, construction, firewood, medicine, and ceremonies and rituals. The use-report does not distinguish between “knowledge” and “real use”, as various potential uses may be known but real use may be different [46]. To account for that, the third section of the questionnaire focused on the real use of the species by asking the informant to score the six use-categories based on the importance of their actual uses. The score varied from 3 (“high use”) to 0 (“not used”) with score 1 for “medium use” and score 1 for “low use” [7]. Informants were additionally asked to rank each plant part based on the importance of their actual uses. Each interview lasted between 2 h and 2 h 30 min.

### Data analysis

#### Knowledge on the uses of *B. aethiopum*

First, for each use-report of *B. aethiopum*, the relative frequency of citation defined as how often a use-report was mentioned was calculated using the fidelity level (FL) [47]. Only significant use-report (with FL > 5%) were reported here.

1 $$ \mathrm{FL}\left(\%\right)=100\times x/n $$

where *x* is the number of informants who mentioned a specific use and *n* is the total number of informants.

Knowledge on uses of *B. aethiopum* was measured using the relative use-value (UV) [7] which is a modified version of the use-value method introduced by [11]. This modified version of UV allows capturing of all the known uses by an individual within and between use-categories [7]:

2 $$ \mathrm{UV}=\sum \limits_{\mathrm{uc}=1}^{n_{\mathrm{uc}}}{\mathrm{UV}}_{\mathrm{uc}}=\sum \limits_{\mathrm{uc}=1}^{n_{\mathrm{uc}}}\sum \limits_{i=1}^n{\mathrm{UR}}_{\mathrm{uc},i}/n $$

where UR_uc_,_*i*_ is the number of use-report mentioned by informant *i* in for a given use-category uc. In our dataset, UR_uc_,_*i*_ varied from 0 to maximum (UR_uc,*i*_) = 6, meaning that the maximum number of use-report mentioned by an informant in a use-category was 6. UV_uc_ is the use-value for a given use-category uc which is the mean of UR_uc_,_*i*_ for that use category; *n*_uc_ is the number of use-categories in the study (*n*_uc_ = 6); *n* is the number of informants.

UV stands as a mean of UR_uc,*i*_ and could vary from 0 to *n* × *n*_uc_ × 6 (in case all informants mentioned all use-categories and that all informants cited a number of use-report equals to 6 in each use-category). Because UR_uc,*i*_ is a count data, a generalized linear model (GLM) with Poisson error distribution [48] was used to assess variation of UV (response variable) with respect to region, local abundance, age category, and gender of informants (predictors). All predictors were categorical with respectively three (humid, sub-humid, semi-arid), two (high, low), three (young, adult, old), and two (women, men) levels. Interaction terms in the model included (i) interaction of region with age category on one hand and gender on the other hand, (ii) interaction of local abundance with age category on one hand and gender on the other hand, and (iii) interaction of age category with gender. Non-significant terms were sequentially removed from the model. Likelihood ratio test was used to assess the goodness of fit of the final model. The deviance-based pseudo-*R*^2^ was also computed to assess the explanatory quality of the final model.

#### Cultural importance of *B. aethiopum*

The cultural importance of *B. aethiopum* was assessed using the importance index (IP) adapted from [49]:

3 $$ \mathrm{IP}=\sum \limits_{\mathrm{uc}=1}^{n_{\mathrm{uc}}}{\mathrm{IP}}_{\mathrm{uc}}=\sum \limits_{\mathrm{uc}=1}^{n_{\mathrm{uc}}}\sum \limits_{i=1}^n{\mathrm{S}}_{i,\mathrm{uc}}/n $$

S_*i*,uc_ is the score of importance attributed by informant *i* (*i* = 1,…, *n*) for the use-category uc; *n*_uc_ is the number of use-category (*n*_uc_ = 6). IP is the overall importance value of *B. aethiopum* and IP_uc_, the importance value of the use-category uc of *B. aethiopum*.

Values of IP and IP_uc_ vary from 0 to 18 (in case all informants scored all six use-categories as “high use”) and 0 to 3 (in the case all informants scored the use-category uc as “high use”) respectively, with higher values indicating higher cultural importance. Ordered logistic models were used to model the effect of age category, gender, local abundance, and region on the variation in the use importance score of *B. aethiopum*. Backward elimination as described in [50] was used to select the most parsimonious models.

All statistical analyses were performed in the R software v.3.3.2 [51]. Package *fmsb* [52] was used to compute Naglekerke’s pseudo-*R*^2^. Ordered logistic models were run using the function clm2 within the package “ordinal” [53].

## Results

### Diversity of uses

Countrywide, 121 different use-reports of *B. aethiopum* were recorded as follows: medicine (66), handcraft (16), food (16), construction (12), firewood (6), and ceremonies and rituals (5). However, only 28 of all those use-reports were found to be significant (Fidelity Level > 5%) (Table 1). The significant use-reports included 9 food uses (human), 6 construction uses, 5 medicinal uses, 5 handcraft uses, 2 firewood uses, and 1 ceremonies and rituals’ use indicating that consensus was high on food uses than the other uses, medicinal uses being the use-category where the least consensus was observed (only 5 significant uses out of 66 uses) (Table 1).

**Table 1:** Significant use-report of *B. aethiopum* per plant part and use category: processing method, forms of use, purpose of use and fidelity level (FL) per chorological region (Hu = Humid, Sub-hu = Sub-humid, Sem = Semi-arid) with illustrations on Fig. 3. Only uses with FL ≥ 5% in at least one region are displayed

Plant parts	Use category	Processing method	Form of use	Purpose of use	Chorological regions		
					Hu	Sub-hu	Sem
Fruits	Food	Remove the flesh	Eat	Human food	2.77	66.02	82.61
		Boil in water the fruits			86.15	4.21	6.21
		Boil in water fruits with corn (maize, rice or millet)			–	2.59	7.14
		Toast fruits			–	–	30.75
	Medicine	Soak fruits in cold/boiled water	Drink the liquid	Malaria	8.31	5.50	–
	Other uses	Collect fruits	Set around or in house, room	Discard shrews and snakes	2.77	47.57	–
Hypocotyle	Food	Boil hypocotyls	Eat	Human nutrition	32.41	66.99	85.16
		Toast hypocotyls			–	1.94	29.19
	Medicine	Soak in alcohol	Drink the liquid	Stomach-ache	1.11	43.37	–
Leaves	Handcraft	Harvest leaves from juveniles and weave (without the limb)	Fan	Home-use	37.95	82.85	4.04
			Mat		0.28	15.86	4.04
			Hat	Clothing	2.49	24.60	–
		Harvest leaves from trees and bind	Sweeping	Home-use	–	–	20.50
	Construction	Harvest leaves from trees	Use with stems to enclose the house	Palissad	0.55	6.15	–
			Cover roof of house	Roof	11.63	22.98	–
Stipe	Medicine	Recuperate the sap after logging a tree	Rinse the mouth	Tooth decay	–	–	13.35
	Construction	Cut down trees and split longitudinally	Chevron	House construction	47.65	–	13.35
			Beam		–	39.16	0.31
			Post		–	17.15	–
Petiole	Handcraft	Crush fresh petiole bark	Use as sponge	Home use/trade	–	15.86	–
	Construction	Collect petioles	Use petioles as girder in poultry house	Home-use	0.28	1.94	9.01
	Firewood	Collect dried petioles	Firewood	Home-use	45.71	14.24	29.50
Seeds	Food	Break nuts of green fruits using rocks and extract the almond	Snack food	Human nutrition	6.37	0.97	5.90
		Break nuts of green fruits using rocks and take the liquid			8.86	0.32	5.90
		Let seeds germinate and extract the almond			–	–	12.73
	Firewood	Collect dried husk of seeds	Firewood	Home-use	1.66	–	5.90
Roots	Medicine	Soak roots in water	Take a bath	Tonify infants	–	7.77	–
		Boil in water	Drink the liquid	Malaria	2.77	5.83	–

The number of significant use-reports per plant part was higher for fruits (6 uses) and leaves (6 uses). Stem was involved in four uses while seeds, hypocotyles, and petioles were each involved in three significant use-reports. Roots had only two significant use-reports (Table 1).

**Fig. 3:**
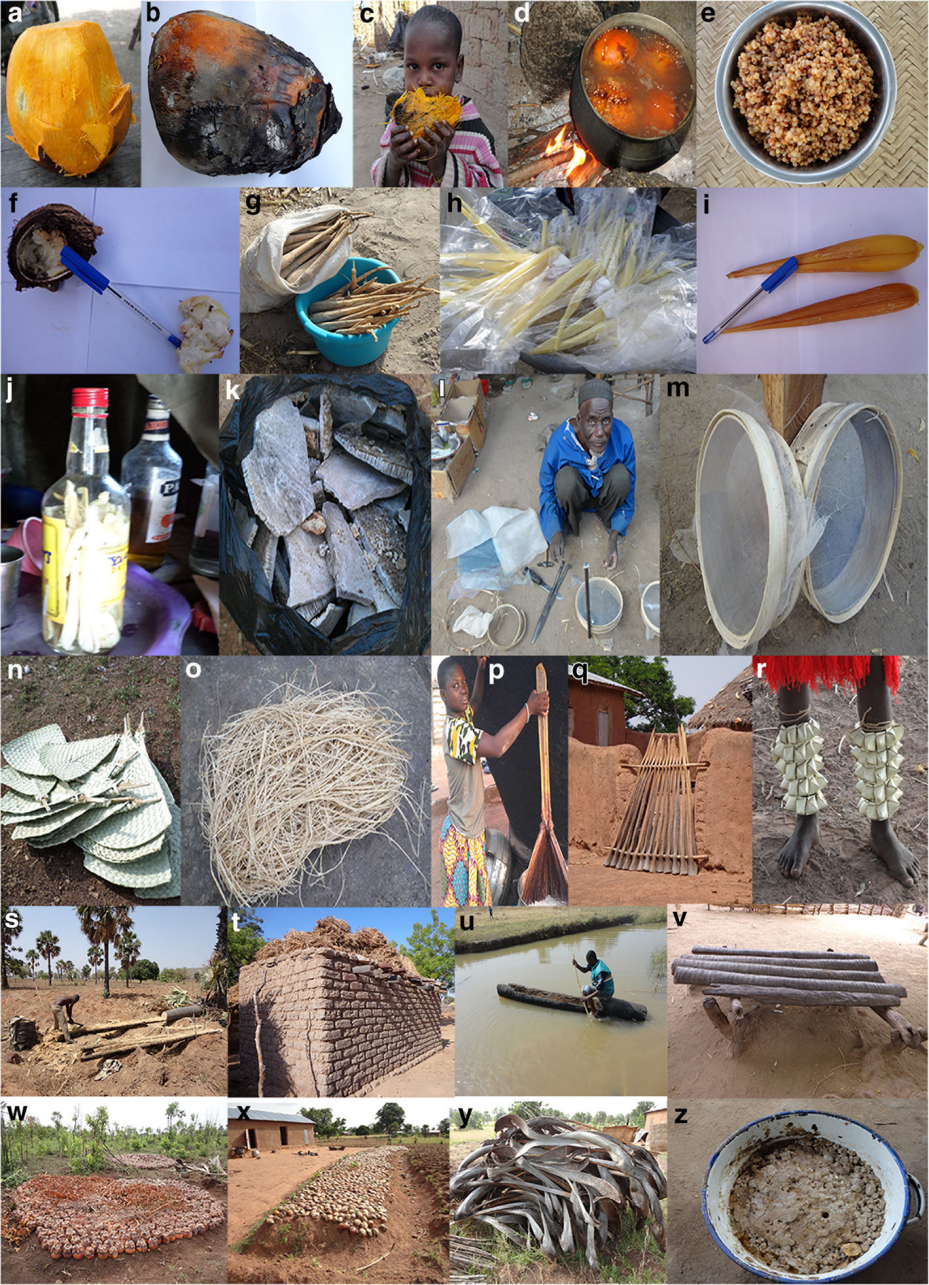
Illustrations of some use-reports of *B. aethiopum* in Benin. **a** Fruits with removed flesh. **b**, **c** Toasted fruits. **d**, **e** Boiling the ripen fruits in water with corn (maize, rice or millet). **f** Almond after germination. **g** Freshly harvested hypocotyls. **h** Boiled and packaged hypocotyls ready for sale. **i** Boiled hypocotyls not cut yet. **j** Fresh hypocotyls cut and put in palm alcohol. **k** Solid potash from incinerated seed hulls. **l** An old man making sieve. **m** Samples of sieves. **n** Fans made from leaves. **o** Sponge made from petioles. **p** Battledore from petioles. **q** Gate from petioles. **r** Implement made of leaves for ceremonies in *Berba* region. **s** A farmer logging a male tree. **t** Stem used in construction. **u** Canoe made from stem. **v** Seat made from stem at public places. **w** Fruits sowed on farm for hypocotyls production. **x** Fruits sowed at home for hypocotyls production. **y** Petioles stored for firewood. **z** Soap “koto” made from seeds hull. Credits to Salako et al. [30]

Uses of plant parts of *B. aethiopum* (see Fig. 3) varied across regions. Fruits and leaves had multiple forms of use, some uses being reported by more than 50% of the informants. These two plant parts in addition to hypocotyl were involved in uses with the highest fidelity level, up to 86% in some regions. Seed uses were relatively not common, with fidelity level less than 15%. Use of root was mentioned only in the humid and sub-humid regions where it is used either to treat malaria or to strengthen children (Table 1). Processing methods were also not similar across regions (Table 1). For example, ripe fruits are toasted (Fig. 3b, c) only in the semi-arid region, while boiled (Fig. 3d) in the humid region before consumption (Table 1). In the semi-arid region, fruits are often boiled in association with cereals (either maize or millet or sorghum) (see Fig. 3e). The hypocotyles were consumed either boiled (Fig. 3g–i) (in all regions) or toasted (only in the semi-arid region) (Table 1). The use of fruits to discard shrews and snakes from homestead or chicken coop was essentially reported in the sub-humid region. There was no mention of this use in the semi-arid region. Many handcraft products (e.g., fan, mat, hat, sponge; see Fig. 3l–q) are made from leaves and petioles of *B. aethiopum*. It is worth noticing that other plant parts including bark and flowers of male and female trees were involved in medicinal and ceremonies and rituals uses that were not significant (FL < 5%), hence not presented here.

**Fig. 4:**
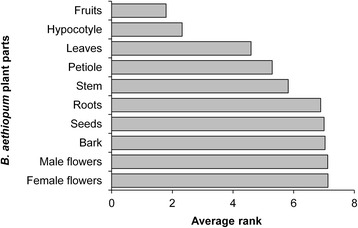
Average actual use rank of plant parts of *B. aethiopum*

Based on the ranking of plant parts with respect to their importance for informants, fruit was ranked first and was followed by hypocotyl (Fig. 4) due to their food uses and commercial value. These two plant parts were the most sold on the local market either in rural or urban areas mainly by women and children. Fruits are collected from the wild. Hypocotyles are either collected from the wild or harvested from fruits sown on farm or at homestead (Fig. 3w–x). Apart from fruits and hypocotyles, the following plant parts were successively leaves, petioles, and stem (Fig. 4).

### Use-value of *B. aethiopum*: effect of region, local abundance, gender, age category, and their interactions

#### Main effect of local and regional abundance, gender, and age category on overall and by use-category UV

There was a significant (*p* < 0.05) relationship between overall knowledge of *B. aethiopum* uses and age category, gender, local abundance and region, either as main effect or in a significant interaction term (Table 2). Informants in the drier regions have more knowledge on the species use. Informants in the humid region reported less uses (mean ± standard error; 3.21 ± 0.08) than in the sub-humid (5.32 ± 0.14) and semi-arid (4.07 ± 0.11) regions. The younger the interviewee is, the lesser he has knowledge on the species uses. Young informants reported less uses (3.80 ± 0.11) than adults and old informants who reported similar number of uses, 4.36 ± 0.11 and 4.24 ± 0.14, respectively. Men (4.31 ± 0.10) were more knowledgeable than women (3.98 ± 0.10). Informants from areas with high local abundance reported more uses (4.19 ± 0.09) than informants from areas with low local abundance (4.09 ± 0.10).

**Table 2:** Predictors in the best Poisson and ordered logistic models showing the relationship between socio-demographic attributes (gender and age category), regional (Region) and local abundance, with use-value and importance index, respectively for *B. aethiopum* (−, non-significant term)

Predictors	Use-value			Importance index		
	df	dev.	*p* value	df	dev.	*p* value
Region (R)	2	177.44	< 0.001	2	353.95	< 0.001
Local abundance (A)	1	0.01	0.915	1	2.22	0.137
Age category (G)	2	17.54	< 0.001	2	10.74	0.005
Gender (S)	1	6.36	0.012	1	4.57	0.045
R × G	–	–	–	4	9.20	0.036
A × G	2	8.94	0.011	–	–	–
G × S	–	–	–	2	5.41	0.049
Goodness of fit test (Error)	981	1053.66	0.753	976	2154.52	0.999
*R*-square (%)	36.96			34.80		

*df* degree of freedom, *dev.* deviance, *p value* probability value computed from a Chi-square distribution

**Fig. 5:**
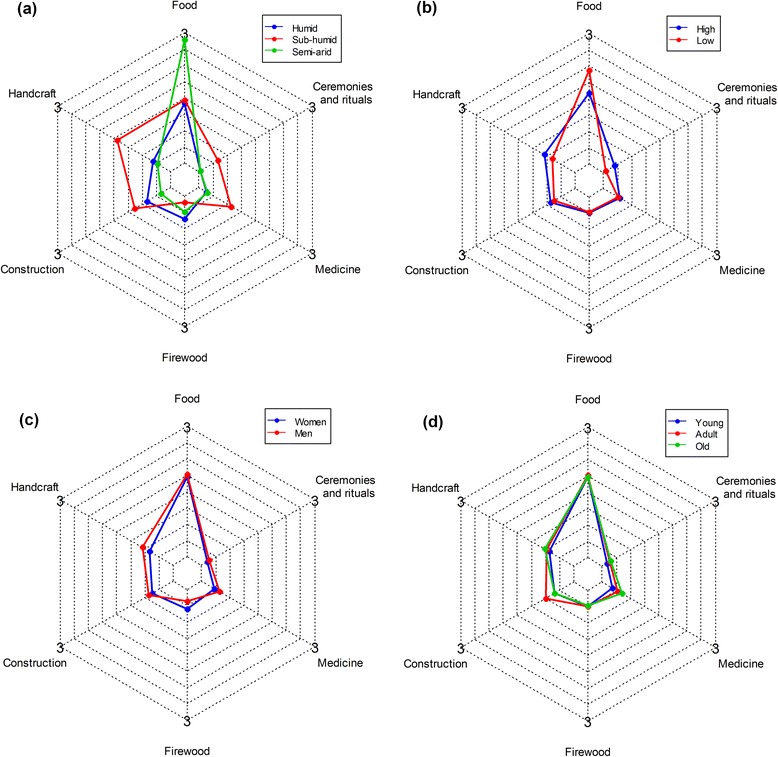
Radar chart showing the main effect of region (**a**), local abundance (**b**), gender (**c**), and age category (**d**) on the UV of *B. aethiopum* across use-categories

Knowledge on the uses of *B. aethiopum* varied greatly across use-categories (Fig. 5). Irrespective of the examined factors, knowledge was higher for food use (1.87 ± 0.04) followed successively by handcraft (0.72 ± 0.03), construction (0.60 ± 0.03), medicinal (0.41 ± 0.02), firewood (0.36 ± 0.02), and ceremonies and rituals uses (0.18 ± 0.01) (Fig. 5). Knowledge on food use was higher in the semi-arid region than in the sub-humid and humid regions that had similar knowledge (Fig. 5a). Knowledge on medicinal, construction, and handcraft uses were higher in the sub-humid region than in the other regions (Fig. 5a). Knowledge on food use was higher in areas with low local abundance while higher in areas with high local abundance for handcraft and ceremonies and rituals uses (Fig. 5b). Regarding gender, men always reported more knowledge than women irrespective of the use-category except for firewood use where the UV was higher for women (Fig. 5c). With respect to age category, knowledge was always lower for young than adults and old informants who had similar knowledge, except that for the food uses, the differences were relatively narrower than for the other use-categories (Fig. 5d).

#### Interacting effect of region and local abundance with socio-demographic attributes

**Fig. 6:**
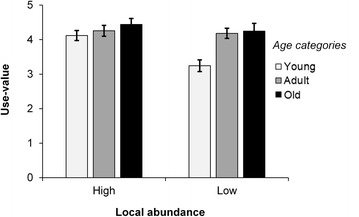
Interacting effect of age category and local abundance on the UV of *B. aethiopum*

Among socio-demographic attributes, only age category was involved in significant interactions with local abundance (Table 2). Young, adult, and old informants had similar knowledge in areas with high local abundance while greater differences were observed in areas with low local abundance, in particular between young and both adult and old informants who had similar knowledge (Fig. 6).

### Cultural importance of *B. aethiopum*

#### Main effect of region, local abundance, gender, and age category on the overall and by use-category importance

There was a significant (*p* < 0.05) relationship between overall importance (IP) of *B. aethiopum* and region, local abundance, gender and age category; either as main effect or in interacting effect (Table 2). *B. aethiopum* was more important (higher IP) in the sub-humid region (mean ± standard error; 5.65 ± 0.08) than in the semi-arid (4.54 ± 0.08) and humid (3.51 ± 0.13) regions; slightly more important for men (4.52 ± 0.10) than for women (4.50 ± 0.09); more important in areas of high local abundance (4.55 ± 0.08) than in area of low local abundance (4.46 ± 0.10); and more important for adults (4.71 ± 0.10) and young (4.44 ± 0.12) than old informants (4.34 ± 0.12).

**Fig. 7:**
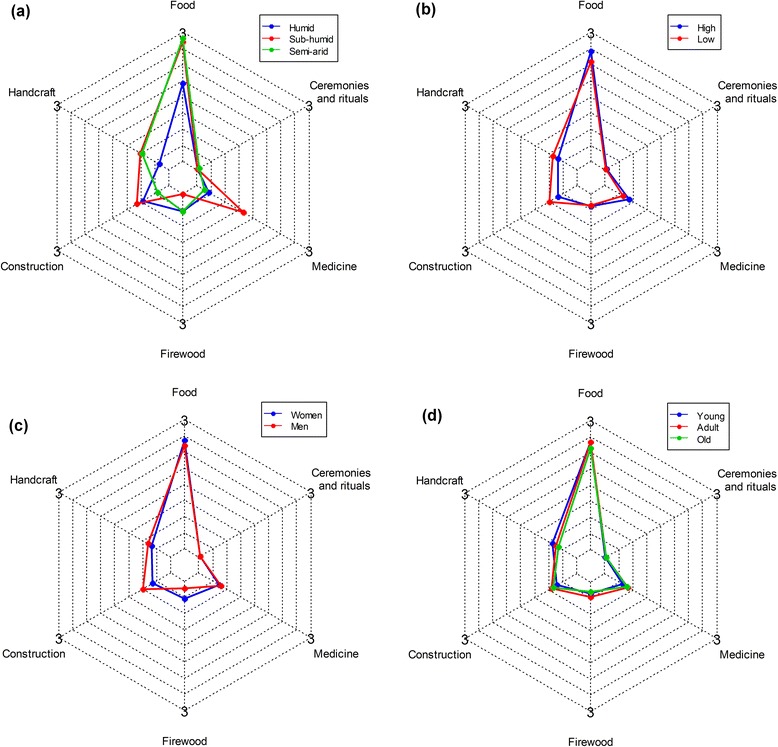
Radar chart showing the main effect of region (**a**), local abundance (**b**), gender (**c**), and age category (**d**) on the IP of *B. aethiopum* across use-categories

The IP varied greatly among use-categories and for each of the examined predictors (Fig. 7). Irrespective of the predictors, food use was the most important (2.45 ± 0.03) followed by construction (0.61 ± 0.03), handcraft (0.57 ± 0.03), and medicinal (0.56 ± 0.03) uses and then firewood (0.29 ± 0.02) and ceremonies and rituals uses (0.03 ± 0.01) (Fig. 7). Considering region, food use was more important in the semi-arid and sub-humid regions than in the humid region (Fig. 7a). Medicinal use was more important in the sub-humid region while the firewood use was roughly not important there (Fig. 7a). Considering local abundance, food and medicinal uses were more important in areas with high local abundance than in areas with low local abundance (Fig. 7b). Handcraft and construction uses were more important in areas with low local abundance than in areas with high local abundance (Fig. 7b). With respect to gender, food and firewood uses were more important for women than men while handcraft use was more important for men than women (Fig. 7c). Regarding age categories, food use was more important for young and adult than old informants. Contrary to food use, medicinal and construction uses were more important for adults and old informants than young (Fig. 7d).

#### Interacting effect of region and local abundance with socio-demographic attributes

**Fig. 8:**
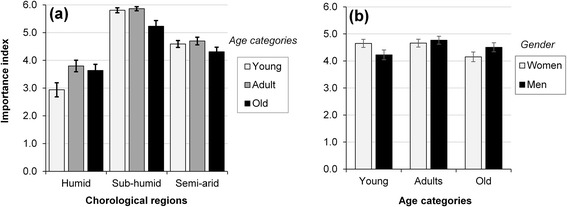
Interacting effect of region and age category (**a**) and gender and age category (**b**) on the IP of *B. aethiopum*

There was no significant interaction involving local abundance and either socio-demographic attributes. However, significant effect was observed for the interaction of region and age category for the overall importance of *B. aethiopum* (*p* = 0.036; Table 2). *B. aethiopum* was more important for young and adults in the sub-humid and semi-arid regions while less important for young in the humid region (Fig. 8a). There was also significant interaction of gender and age categories (*p* = 0.047; Table 2). Accordingly, *B. aethiopum* was relatively more important for young women than young men, similarly important for adult men and women while relatively more important for old men than old women (Fig. 8b).

## Discussion

This study reported on the relationships between traditional knowledge (TK) and cultural importance (CI) of *B. aethiopum* on one hand, and informant socio-demographic attributes (age categories and gender) on the other hand, considering the ecological apparency hypothesis (EAH) at both local and regional scale. It was found that TK and CI of *B. aethiopum* varied greatly across use-categories: first, food use and then successively handcraft, construction, medicinal, firewood, and ceremonies and rituals uses (1). Significant difference was also observed among age categories, younger informants reporting less uses than adults and old informants who reported similar number of uses (2). Men reported more uses than women (3). Local abundance had significant effect on TK and CI: informants in areas of higher abundance reported more uses and higher score than informants in areas of lower abundance (4). Also, regional abundance determined TK and CI, region of low abundance (humid region) reporting less uses and lower score than region of larger abundance (sub-humid and semi-arid regions) (5). It was also found that local abundance influences relationships between TK (respectively CI) and age categories: young, adult, and old informants having similar knowledge in areas of high local abundance while greater differences were observed in areas of low local abundance in particular between young and both adult and old who had similar knowledge (6).

### Interacting effect of age categories, gender, local abundance, and region as drivers of TK

Our data support the general trend that TK depends on age categories and gender [4, 54, 55]. Young informants often reported less uses than adults and old informants, hence congruent with the assumption that TK is a time-dependent process of learning [6]. Therefore, informants from older age category, having spent a longer time with their natural environment, would normally have more knowledge than informants from younger age category [4]. Although women are reputed to have more contacts with NTFPs than men [56], the fact that men often have more knowledge than women in our case study may be because women are often specialized in some uses of a species, most often food and to some extent medicinal uses while men uses expand to other uses (e.g., construction, ceremonies and rituals). This utilization pattern of resources is often dictated by differences in activities and roles of men and women within households [54] and sometimes to cultural taboos or prohibitions [7].

The EAH has multiple implications in conservation biology. For example, a positive relationship between species visibility and their use imply gradual elimination of the more apparent species by predatory collection through the constant pressure for domestic/commercial use [17]. The finding that TK was globally higher in areas of higher apparency (either local or regional) supports the ecological apparency hypothesis. Previous studies using abundance as a quantitative predictor of UV have come to similar conclusions [57, 58]. However, others found either no link or at best a weak relationship [15, 16]. Therefore, the EAH would not always explain pattern of uses of species, and other aspects such as socio-cultural, economic, and political aspects should be accounted for [14] and may explain the failure of our model to capture about 60% of the variation in TK. Indeed, human decision processes are complex and cannot be reduced to only ecological or economic considerations; other factors such as social and cultural are important in the processes involved in choosing and harvesting a plant [59].

Informants in drier regions reported more uses than informants in the wettest region which is congruent with the EAH prediction at the regional scale following the regional pattern of *B. aethiopum* across the country: *B. aethiopum* abundance and distribution is proportional to dryness, increasing northwards (from the wettest to the driest region) [30]. In Benin, previous studies have shown that diversity of wild edible plant species and plant in home gardens declines towards the semi-arid region [60–62], suggesting a likely higher intensity of use on a narrow number of species when the climate becomes drier which may in turn results in more knowledge on the resource at least as food use is concerned. At a local scale, the EAH also overall proved true: the higher the local abundance, the higher the knowledge informants have on the species. However, looking at patterns within each region (not statistically tested because of lack of replicates), this seems to not be the case in the semi-arid region where informants in the village of low local abundance reported up to two times more uses than informants in the village of high local abundance (low abundance: 5.50 ± 0.14 use-reports, high abundance: 2.89 ± 0.09 use-reports), compared to the sub-humid (low abundance: 3.70 ± 0.21 use-reports, high abundance: 6.49 ± 0.13 use-reports) and humid regions (low abundance: 3.24 ± 0.14 use-reports, high abundance: 3.19 ± 0.10 use-reports). As such, differences in TK due local apparency could be related to regional apparency. This pattern suggests two hypotheses. First, in the village of low local abundance in the semi-arid region, *B. aethiopum* was abundant in a recent past and likely has undergone rapid decline as reported by informants in that village (Additional file 3). This thus raises the importance of understanding the species past abundance in understanding current patterns of knowledge people have on them. Second, in the village of high abundance, the spread of uses of *B. aethiopum* is narrowed because other species fulfill the role of *B. aethiopum* observed in the village of low local abundance as suggested by the diversification hypothesis. The diversification hypothesis considers that the presence of multiple species in an environment amplifies the spectrum of alternatives [63] for subsistence, health, and livelihoods in general, thus reducing the pressure (use) on a single species, hence knowledge of its use. Apart from the regional apparency, one additional factor that may explain between regions differences for a given local abundance (e.g., considering villages of high abundance) is differences in ethnic affiliations [61, 64, 65]. Different ethnic groups have often different life style, beliefs, and perceptions of their environment that translate in different knowledge of resources of their environment [64] as previously reported for many other species in Benin [65–67].

This study also provides empirical evidence that pattern of knowledge distribution across age categories depends on local and regional apparency of the studied resources as we predicted. The most common (locally or regionally) a species is, the most likely knowledge on its utilization is similar across age categories. In contrast, the less common a species is, the relatively greater is the gap of knowledge among age categories, especially between young on one hand and adult and old people on the other hand. While knowledge acquisition as time-dependent process [6] is straightforward in explaining the often lower knowledge of younger informants as compared to older informants, local or regional apparency may provide interesting potential explanation of the magnitude of the gap of knowledge among age categories. Our proposition is that higher local or regional apparency seems to speed the process of knowledge acquisition resulting in similar knowledge among age categories. At the opposite side, lower local or regional apparency slow the process of knowledge acquisition and therefore result in greater discrepancies among age categories, in particular between younger and older informants. Additional studies on others species would be needed to clarify this proposition.

### Cultural importance of *B. aethiopum*

Understanding the cultural importance of plant resource is crucial for an informed management [7]. Differences in form of uses across regions (see Table 1) are mostly linked to cultural differences due to different ethnic affiliations. For example, Gourmantché people from the semi-arid region toast the fruit before consumption. Such use was not mentioned in the other regions.

Most culturally important uses of *B. aethiopum* in study regions were successively food, handcraft, construction, and medicine. These use-categories are also the most known and important for palm species in Latin America [68, 69]. Irrespective of regions, local abundance, gender, and age categories, food use was the most culturally important, clearly indicating that *B. aethiopum* is primarily a food palm species in Benin, particularly in the sub-humid and semi-arid region. This corroborates Assogbadjo et al. [60] who reported *B. aethiopum* as a priority wild edible tree species in these two regions.

The finding that food uses was more important for women than men confirms the previous hypothesis of women specialization in the food use category but also stress on the relationship between patterns of plant uses and activities/roles in African households [54]: women are responsible for kitchen and most often are the sellers of food products in markets. This is further confirmed by the greater value of importance index for use-categories handcraft, construction and medicinal for men than women since culturally, men are often responsible for constructions and health care of the household members [70].

**Fig. 9:**
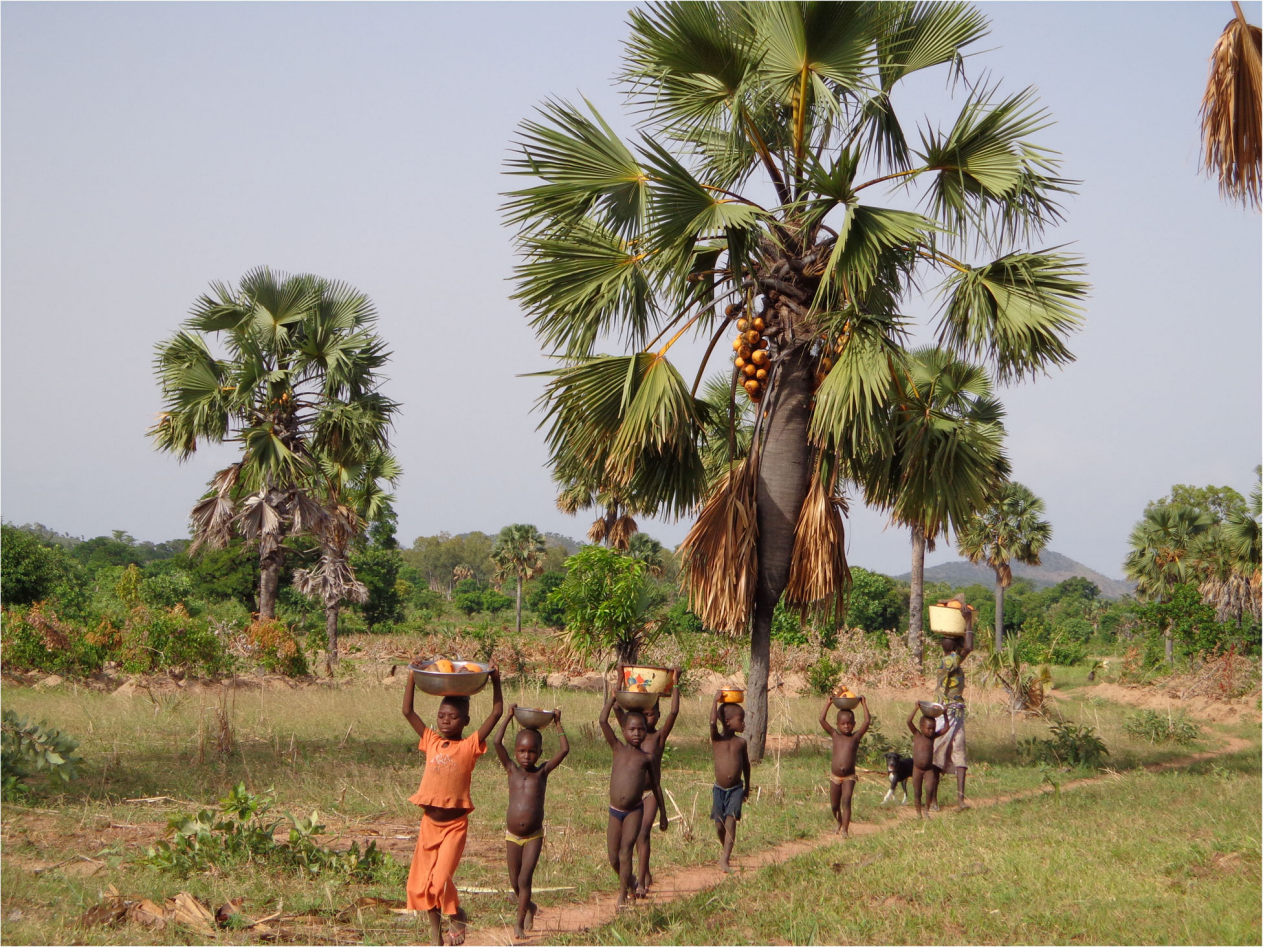
An old women and children on the way back to home from collection of *B. aethiopum* fruits in northern Benin. Credits to Salako et al. [30]

The fact that fruits and hypocotyles were the most important plant parts added to its greater commercial value are an additional evidence that *B. aethiopum* is a food palm species. Moreover, the greater commercial value of these two plant parts for women than men confirms *B. aethiopum* as a “women-palm” species (Fig. 9) as also reported in Brong Ahafo region in Ghana [35]. However, this great cultural and commercial value of fruits and hypocotyls if not well controlled may reduce regeneration potential in natural stands of *B. aethiopum*, threatening its population rejuvenation as reported for other species, e.g., *Pentadesma butyracea* [71].

Leaves were the second plant part with the highest number of significant use-report and the third most important plant part. They were often harvested from saplings and juveniles because adults are often taller (up to 20 m, see) and it is not easy to climb [30]. As reported in other studies [25, 72], stipe of *B. aethiopum* is a much appreciated material for construction and likely explain why it was so culturally important in the study regions. For example, roughly all houses in the village Loumbou-loumbou are made of *B. aethiopum* stipe (Salako, field observations; Fig. 3t). Gamba Begounou, an 83-year-old women, said “*B. aethiopum* is the only one good tree for house construction here. It is very resistant and can lives more than two hundred years”.

Medicinal use of *B. aethiopum* was also culturally important for surveyed informants and aligned with [35] who reported its medicinal use in 14 out of 28 African countries where it occurs and as the third most used palm species in traditional medicines in Africa. However, in spite of having four times more uses than food use-category (66 versus 16), only 5 were significant, suggesting a lack of consensus on most of the medicinal uses. This is likely due to the fact that the medicinal uses were mostly ethnic-specific. The common use of fruits against malaria also consensually reported in this study has been recently confirmed by pharmacological prospects [35].

Surprisingly, the use of sap commonly reported for *B. aethiopum* in other countries (e.g., Cote d’Ivoire, Senegal and Guinea; see [28, 32, 33]) was not mentioned in our study. In the humid region, this may be due to the fact that people reputed for palm wine extraction preferred *Elaeis guineensis* which is the ancestral source of palm wine in Benin [73]. In the sub-humid and semi-arid regions where *E. guineensis* is roughly absent, this may be due to (but not limited to) the fact that palm wine extraction is not their habit and that they do not have such knowledge. However, the use of fruits to discard shrews and snakes appears as a “new” reported use for *B. aethiopum*. This property may have potential for biological control of pests and hence required phytochemical screening prospection.

## Conclusions

Dearth of information on traditional knowledge and cultural importance of species has been implicated for their non-sustainable utilization. This study confirms that traditional knowledge is closely linked to gender and age but provides additional evidence that this relationship is further influenced by local and regional apparency of the resource: greater discrepancies between younger and older informants in areas of lower apparency. We propose that this is linked to the speed of knowledge acquisition which we postulate is lower in areas of lower apparency. Therefore, study reporting on knowledge distribution among age categories should account for the local and regional availability of the study resources in explaining the observed patterns and further shade this by the past abundance of the resource in the study environment. Additional studies on others species are needed to clarify this proposition. This study also showed the paramount local importance of *B. aethiopum* in Benin in particular for people of the sub-humid and semi-arid regions, providing them with fundamental good and services (food, medicine and materials for house construction) with a high potential to generate cash income for women. *B. aethiopum* is therefore a particularly important tree species which deserves more attention than it is currently given. From a management perspective, women should be trained for good practices of fruits and hypocotyls collection to avoid overutilization. From a domestication perspective, further studies should with priority focus on fruits and hypocotyls. As a first step, traditional classification will provide good insights, and because of their specialization, women could provide valuable knowledge. Women also should be of particularly interest when selecting “plus trees” for desired traits in fruits and hypocotyls.

## Additional files

Additional file 1: Socio-demographic attributes (ethnic group, age category and gender) of informants and local names of *B. aethiopum*. (DOCX 13 kb)

Additional file 2: Questionnaire for assessing use-value and cultural importance of *B. aethiopum*. (DOCX 15 kb)

Additional file 3: Local perception on the dynamic of *B. aethiopum*. (DOCX 28 kb)
