# Supplementary material for: Traditional knowledge and cultural importance of Borassus aethiopum Mart. in Benin: interacting effects of socio-demographic attributes and multi-scale abundance
Source: J Ethnobiol Ethnomed. 2018 May 15;14:36. doi: 10.1186/s13002-018-0233-8 (PMC5952639; doi:10.1186/s13002-018-0233-8)
Supplement: Supplementary file 1 — Socio-demographic attributes (ethnic group, age category and gender) of informants and local names of B. aethiopum. (DOCX 13 kb) [file 13002_2018_233_MOESM1_ESM.docx]

**Additional file 1.** Socio-demographic attributes (ethnic group, age category and gender) of informants and local names of *B. aethiopum*

| Regions | Abundance levels (villages) | Ethnic groups | Young | Adult | Old | Total |
| --- | --- | --- | --- | --- | --- | --- |
|  |  |  | Women/Men | Women/Men | Women/Men |  |
| Humid | High (Hounviatoun) | Sahouè | 23/24 | 38/36 | 31/29 | 181 |
|  | Low (Agonkanme) | Fon | 17/22 | 25/22 | 19/26 | 131 |
|  |  | Sahouè | 7/7 | 9/13 | 9/4 | 49 |
| Sub-humid | High (Ouoghi) | Nagot | 27/29 | 37/31 | 24/32 | 180 |
|  | Low (Kprèkètè) | Anan | 18/12 | 3/6 | 1/8 | 48 |
|  |  | Lokpa | 5/7 | 20/19 | 18/12 | 81 |
| Semi-arid | High (Pingou) | Berba | 36/28 | 26/27 | 28/32 | 177 |
|  | Low (Loumbou-loumbou) | Gourmantché | 24/22 | 27/32 | 21/19 | 145 |
