# Supplementary material for: Traditional knowledge and cultural importance of Borassus aethiopum Mart. in Benin: interacting effects of socio-demographic attributes and multi-scale abundance
Source: J Ethnobiol Ethnomed. 2018 May 15;14:36. doi: 10.1186/s13002-018-0233-8 (PMC5952639; doi:10.1186/s13002-018-0233-8)
Supplement: Supplementary file 2 — Questionnaire for assessing use-value and cultural importance of B. aethiopum. (DOCX 15 kb) [file 13002_2018_233_MOESM2_ESM.docx]

**Additional file 2:** Questionnaire for assessing use-value and cultural importance of *B. aethiopum*

**Section 0. Data on the village and region of the informant**

| Region: | Name of the village: |  |
| --- | --- | --- |
| Local abundance level (check): High ⃝ Low ⃝ | | |

**Section 1. Socio-demographic data of the informant**

| 1.1. Identification number: | 1.3. Ethnic group: |
| --- | --- |
| 1.2. Name of the informant: | |
| 1.4. Informant language: | 1.4- Gender: Men ⃝ Women ⃝ |
| 1.5. Age : | 1.6. Length of residence: |

**Section 2. Free-listing of the known uses of *B. aethiopum***

2.1. Do you know this species (showing the species picture)? Yes ⃝ No ⃝

2.2. How do you call it in your language and what is the meaning of this designation?

| Local designation |  |
| --- | --- |
| Meaning of the designation |  |

2.3. Based on your experience in this village, how could you rate the species dynamic trend?

| Stable | Decreasing | Increasing |
| --- | --- | --- |
| ⃝ | ⃝ | ⃝ |

2.4. Please list the uses of *B. aethiopum* you know

| Use-categories | N⁰ | Plant part used | Give the specific use and describe how you use it |
| --- | --- | --- | --- |
|  | 1 |  |  |
|  | 2 |  |  |
|  | 3 |  |  |
| Medicinal  uses | 1 |  |  |
|  | 2 |  |  |
|  | 3 |  |  |
|  | 4 |  |  |
|  | 5 |  |  |
| Handcraft uses | 1 |  |  |
|  | 2 |  |  |
|  | 3 |  |  |
| Construction uses | 1 |  |  |
|  | 2 |  |  |
|  | 3 |  |  |
| Firewood uses | 1 |  |  |
|  | 2 |  |  |
|  | 3 |  |  |
| Ceremonies and ritual uses | 1 |  |  |
|  | 2 |  |  |
|  | 3 |  |  |

**Section 3. Importance of the actual uses of *B. aethiopum***

3.1. Could you please score these six use-categories based on their importance as regard how yourself use *B. aethiopum*. Use the following scale “high use”, “medium use”, “low use”, “no use”

| Use-categories | Food | Medicinal | Handcraft | Firewood | Ceremonies and ritual uses | Construction |
| --- | --- | --- | --- | --- | --- | --- |
| Score |  |  |  |  |  |  |

3.2. Could you please rank the plant parts of the species based on their importance as regard how yourself use *B. aethiopum*.

| Plant parts | Fruit | Hypocotyle | Leave | Petiole | Stem | Root | Bark | Other (specify) | Other (specify) |
| --- | --- | --- | --- | --- | --- | --- | --- | --- | --- |
| Rank |  |  |  |  |  |  |  |  |  |
