# Supplementary material for: Traditional knowledge and cultural importance of Borassus aethiopum Mart. in Benin: interacting effects of socio-demographic attributes and multi-scale abundance
Source: J Ethnobiol Ethnomed. 2018 May 15;14:36. doi: 10.1186/s13002-018-0233-8 (PMC5952639; doi:10.1186/s13002-018-0233-8)
Supplement: Supplementary file 3 — Local perception on the dynamic of B. aethiopum. (DOCX 28 kb) [file 13002_2018_233_MOESM3_ESM.docx]

**Additional file 3.** Local perception on the dynamic of *B. aethiopum*

Information on local perception on the dynamic of *B. aethiopum* was collected from only adult and old informants. Young were not considered because too young to provide relevant information regarding this aspect. Overall, 92 % of adult and old informants (627/684) indicated the dynamic of *B. aethiopum* population in their environment. Significant variation of perceptions was found between areas of low and high local abundance in each chorological region (Fig. A). Informants from areas with low local abundance indicated more often decline (88 % to 100 % of informants, Fig. A) than those in high local abundance (57.25 % to 69.72 %). Stable dynamics was mostly mentioned in areas with high local abundance. Increasing dynamics was also mostly mentioned in areas with high local abundance (Fig. A).


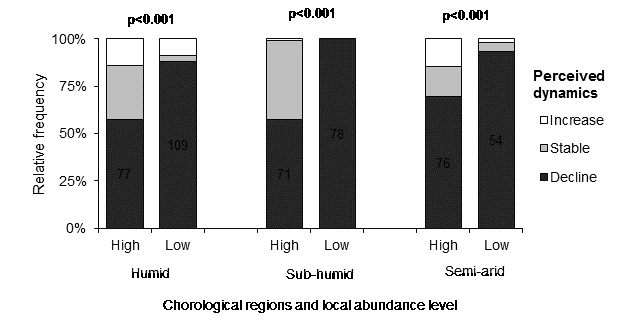


Figure A. Perceptions on the dynamic of *Borassus aethiopum* according to informants (p are p-value from Fisher’s exact test).
